# Infant mortality: a call to action overcoming health disparities in the United States

**DOI:** 10.3402/meo.v18i0.22503

**Published:** 2013-09-10

**Authors:** Allison A. Vanderbilt, Marcie S. Wright

**Affiliations:** 1 Assessment and Evaluation, Center on Health Disparities, School of Medicine, Virginia Commonwealth University, Richmond, VA, USA; 2 Research Support Services, Center on Health Disparities, School of Medicine, Virginia Commonwealth University, Richmond, VA, USA

**Keywords:** health disparities, underserved populations, infant mortality, medical health

## Abstract

Among all of the industrialized countries, the United States has the highest infant mortality rate. Racial and ethnic disparities continue to plague the United States with a disproportionally high rate of infant death. Furthermore, racial disparities among infant and neonatal mortality rates remain a chronic health problem in the United States. These risks are based on the geographical variations in mortality and disparities among differences in maternal risk characteristics, low birth weights, and lack of access to health care.

Infant mortality is a key indicator for the overall health of the population and for health care quality (1). Among all of the industrialized countries, the United States (2, 3) has the highest infant mortality rate (IMR) (4). Furthermore, racial and ethnic disparities continue to plague the United States with a disproportionally high IMR; for example, African American infants have more than double the IMR compared with Caucasian infants (5). These health disparities between ethnic and racial groups continue to grow (6) due to the widening gap in large declines in infant and fetal mortality among Caucasians compared to African Americans (7). This is further exacerbated by the recent findings from Emuren et al. (8), who noted that African American infants are at higher risk of dying compared to Caucasian infants.

Unfortunately, racial disparities among infant and neonatal mortality rates are a chronic health problem in the United States (9). These racial disparities vary across the states with some locations experiencing more significant problems than others (10, 11). These risks are based on the geographical variations in mortality and disparities among differences in maternal risk characteristics, low birth-weights, and lack of access to health care (10). Tyler et al. argue that these geographical variations are due to inconsistencies in reporting, such as fetal death, infant and neonatal morality rates (12) and racial disparities (13).

With this influx of infant mortality across the United States, it is imperative to incorporate health disparities, such as infant mortality and high risk pregnancies, into our medical school curriculum and for our health care professionals; thus providing them with the foundation and exposure to risk factors related to infant mortality prior to entering the health care workforce. This will assure a successful transition into health care settings; hence this is a call to action for physicians, biomedical researchers, and medical schools with the end result focused on decreasing the IMR within the United States.

## References

[1] Stampfel C, Kroelinger CD, Dudgeon M, Goodman D, Ramos LR, Barfield WD (2012). Developing a standard approach to examine infant mortality: findings from the infant mortality collaborative (SIMC). Matern Child Health J.

[2] Hessols NA, Fuentes-Afflick E (2005). Ethnic differences in neonatal and post-neonatal mortality. Pediatrics.

[3] Kissantas P (2008). Ethnic differences in infant morality by cause of death. J Perinatol.

[4] Kochanek KD, Xu J, Murphy SL, Miniño AM, Kung H-C (2011). Deaths: Final data for 2009. National vital statistics reports; vol 60, no 3.

[5] Murphy SL, Xu JQ, Kochanek KD (2012). Deaths: preliminary data for 2010. Natl Vital Stat Rep.

[6] Heron M, Sutton PD, Xu J, Ventura SJ, Strobin DM, Guyer B (2009). Annual summary of vital statistics: 2007. Pediatrics.

[7] Wingate MS, Barfield WD, Petrini J, Smith R (2012). Disparities in fetal death and first day death: the influence of risk factors in 2 time periods. Am J Public Health.

[8] Emuren L, Chauhan S, Vroman R, Beydoun H (2012). Epidemiology of infant death among black and white non-hispanic populations in Hampton roads, Virginia. South Med J.

[9] Tyler CP, Grady SC, Grigorescu V, Luke B, Todem D, Paneth N (2012). Impact of fetal death reporting requirements on early neonatal and fetal mortality rates and racial disparities. Public Health Rep.

[10] Marks JS, Buehler JW, Strauss LT, Hogue CJ, Smith JC (1987). Variation in state-specific infant mortality risks. Public Health Rep.

[11] Wilson Al, Fenton LJ, Musson DP (1986). State reporting of live births of newborns weighing less than 500 grams: impact on neonatal mortality rates. Pediatrics.

[12] Phelan ST, Goldenberg R, Alexander G, Cliver SP (1998). Perinatal mortality and its relationship to the reporting of low-birthweight infants. Am J Public Health.

[13] Wingate MS, Alexander GR (2006). Racial and ethnic differences in perinatal mortality: the role of fetal death. Ann Epidemiol.

